# Current Management of Bone Metastases from Differentiated Thyroid Cancer

**DOI:** 10.3390/cancers13174429

**Published:** 2021-09-02

**Authors:** Satoshi Kato, Satoru Demura, Kazuya Shinmura, Noriaki Yokogawa, Takaki Shimizu, Hiroyuki Tsuchiya

**Affiliations:** Department of Orthopaedic Surgery, Graduate School of Medical Sciences, Kanazawa University 13-1 Takara-machi, Kanazawa 920-8641, Japan; msdemura@gmail.com (S.D.); kazuyashinmura@yahoo.co.jp (K.S.); chakkun1981chakkun@yahoo.co.jp (N.Y.); takaki.shimizu0928@gmail.com (T.S.); tsuchi@med.kanazawa-u.ac.jp (H.T.)

**Keywords:** differentiated thyroid cancer, bone metastasis, metastasectomy, stereotactic radiosurgery

## Abstract

**Simple Summary:**

Patients with bone metastases (BMs) from differentiated thyroid carcinoma (DTC) can live longer than those with BMs from other cancers. BMs from DTC create destructive lesions and easily cause intractable pain and neurological symptoms, including paralysis. These symptoms related to BMs affect mortality directly and indirectly by hampering the application of systemic therapies. Therefore, long-term local control of BMs in patients with DTC is desired, especially in patients with single or a small number of metastases. Local treatments for BMs have recently become advanced and sophisticated in surgery, radiotherapy, and percutaneous procedures. These therapies, either alone or in combination with other treatments, can effectively improve, or prevent the deterioration of, the performance status and quality of life of patients with DTC-BM. Among local therapies, complete surgical resection and stereotactic radiosurgery are the mainstay for achieving long-term control of DTC-BM.

**Abstract:**

After the lung, the skeleton is the second most common site of distant metastases in differentiated thyroid carcinoma (DTC). Patients with osteolytic bone metastases (BMs) from thyroid carcinoma often have significantly reduced performance status and quality of life. Recent advancements in cancer therapy have improved overall survival in multiple cancer subtypes, including thyroid cancer. Therefore, long-term local control of thyroid BMs is desired, especially in patients with a single metastasis or oligometastases. Here, we reviewed the current management options for DTC-BMs and especially focused on local treatments for long-term local tumor control from an orthopedic tumor surgeon’s point of view. Metastasectomy and stereotactic radiosurgery can be performed either alone or in combination with radioiodine therapy and kinase inhibitors to cure skeletal lesions in selected patients. Percutaneous procedures have been developed in recent years, and they can also have a curative role in small BMs. Recent advancements in local therapies have the potential to provide not only long-term local tumor control but also a better prognosis.

## 1. Introduction

Differentiated thyroid carcinoma (DTC) is the most common endocrine malignancy [1]. The prognosis of DTC is generally favorable, with a 10-year survival rate of over 95% [2,3]. However, in 5% to 25% of patients, distant metastases are detected at the time of diagnosis or during the disease’s course. In patients with DTC, bone metastases (BMs) occur in 2% to 13% of all patients and in nearly half of the patients with distant metastases [2]. In patients with DTC, the bone is the second most common site for distant metastases after the lung [2,4]. The spine is the site where DTC-BMs are most likely to occur, and it is affected in almost half of patients with DTC-BM [5]. BM from DTC is associated with a worse overall prognosis than lung metastasis [6,7,8,9]. However, the prognosis of patients with BM from DTC is still favorable, with a 10-year overall survival (OS) rate of 35% to 47% [10,11], compared with that of patients with BM from other cancers. Despite this relatively favorable prognosis, patients with osteolytic BMs from DTC often have a significantly reduced performance status (PS) and quality of life (QOL), with intractable pain, neurological symptoms, and increased mortality [12,13,14]. Farooki et al. have reported a 78% occurrence of at least one clinical skeletal-related event (SRE) with a median of 5 months from the identification of BM to the first SRE in patients with DTC with BM. After a median of 10.7 months, 65% of patients sustained a second SRE [12]. Importantly, mortality is significantly higher in patients with BM who develop SREs [12]. The goals of treatment for BMs remain palliative, striving toward symptom palliation, and improved PS and QOL, besides the long-term local control of the tumor. Recent advancements in cancer therapy have dramatically improved OS across multiple cancer subtypes. Therefore, long-term local control of thyroid BMs is desired, especially in patients with a single metastasis or oligometastases, who are expected to live longer.

Here, we reviewed the current management options for DTC-BMs and especially focused on local treatment for long-term local tumor control, including surgical metastasectomy, from the orthopedic tumor surgeon’s point of view.

## 2. Systemic Therapy

### 2.1. Radioiodine Therapy

Radioactive iodine (RAI) therapy is the first-line treatment in patients with DTC and RAI-avid metastases [1]. However, RAI is ineffective for larger metastases, although it can extirpate small lesions [15]. RAI refractoriness in DTC metastases has a negative effect on prognosis [7,16].

In treating DTC-BM, RAI therapy is effective for patients with RAI-avid lesions [17], and such patients have a better prognosis than patients with non-RAI-avid lesions [11]. A recent retrospective study reported that RAI therapy in combination with one or more local or systemic therapies was associated with a better prognosis compared with RAI therapy alone [18]. However, this therapy was less effective for BM than for metastases in other organs. It was reported that patients with lung metastases had higher remission rates (50% to 74%) than those with BM (10% to 17%) [4,19]. Moreover, more than 20% of BMs do not show any RAI uptake [4,20].

Patients with small BMs that are undetectable on ordinary image inspections but that are detected on ^131^I diagnostic scans have a better prognosis than patients with large and symptomatic BMs [21]. Generally, large BMs are refractory to ^131^I and cause the occurrence or impending occurrence of SREs. Therapy is insufficient for multiple BMs; other treatment approaches are required [2,21]. RAI therapy may be contraindicated in patients with large BMs in the cranium or spine. This is because the enlargement of the tumor lesions can be induced by increased thyroid-stimulating hormone (TSH) levels, following either the administration of recombinant human TSH or hormone withdrawal, which can lead to compressive symptoms [22]. Specifically, in patients with BMs of the spine, pathological fractures and spinal cord compression from spinal lesions severely compromise PS. A reduced PS in patients with metastatic disease affects mortality directly and indirectly by hindering the delivery of systemic therapies, including radioiodine therapy. For patients with oligometastases, long-term control of large and symptomatic BMs by other treatment options, including metastasectomy, is ideal for prolonged survival and the future application of RAI therapy for other, newly-developed organ lesions. For patients with coexisting vital organ metastases and a large BM, the efficacy of RAI therapy for vital organ metastases can significantly increase after metastasectomy for BM by decreasing the total volume of the tumors.

### 2.2. Kinase Inhibitors

Kinase inhibitors (KIs) were recently applied in the treatment of progressive RAI-refractory DTC with distant metastases, and they offered a favorable outcome [23]. The latest guidelines recommend systemic treatment for patients with progressive RAI-refractory disease and greater tumor burden [1,16,24].

In contrast, in cases of BM, several studies have reported a worse response to treatment and a shorter progression-free survival (PFS) rate among patients treated with sorafenib and sunitinib [25,26,27,28]. In a retrospective study to evaluate KI therapies for DMs from DTC, bone and pleural lesions were the most refractory to therapies [28]. A prospective study showed that the absence of BM independently predicted superior PFS and OS in patients with RAI-refractory DTC who were treated with sorafenib [29]. The BMs that had received external beam radiotherapy (EBRT) before the onset of KI therapy were more susceptible, whereas non-irradiated BMs showed progression despite the response to KI that was shown in non-BM lesions [25]. The progression of BM while on KI may occur despite the sustained benefit of KI at other metastatic sites. These findings indicate that KI therapies alone play a limited role in the treatment of BMs from DTC. The findings also suggest that, for patients with DTC-BM, a multimodal approach should be combined with local and systemic therapies, including KI therapy, which should be used for reducing systemic tumor burden.

### 2.3. Antiresorptive Therapies

Bisphosphonate therapy is the current standard of care for preventing SREs in patients with BM [30,31]. Bisphosphonates inhibit osteoclast-mediated bone resorption and have antitumor effects by inhibiting tumor cell proliferation, adhesion, and invasion; by inhibiting angiogenesis; and by inducing apoptosis [31]. Recently, denosumab, a monoclonal antibody to the receptor activator of nuclear factor-kappa B ligand (RANKL) that inhibits osteoclast activity. It has been frequently used in cases of BM, and it has proven superior to bisphosphonate zoledronic acid in the prevention of SREs [32].

The number of studies that examine the effects of antiresorptive therapy in patients with DTC-BM is still limited. Recent studies have reported that in patients with multiple thyroid BMs, treatment with bisphosphonates can improve QOL and reduce SREs [33,34,35]. Despite the occurrence of BMs, OS in DTC is often significantly better than in other cancers. The potential benefit of antiresorptive therapy in reducing SRE should be weighed against the adverse events associated with its long-term use, such as osteonecrosis of the jaw (ONJ) and atypical femoral fractures. There are no differences between the incidence rates of these adverse events in patients using bisphosphonates and those using denosumab [36,37]. Because patients with malignancies treated with chemotherapy or head and neck EBRT have a higher risk of ONJ, these patients have to undergo a careful dental evaluation before the start of antiresorptive therapies [30]. The potential harm and benefits of combination therapy with antiresorptive drugs and KIs should be verified because anti-angiogenic KI therapies have also been associated with ONJ without antiresorptive therapy in a patient with DTC [38].

## 3. Local Therapy

### 3.1. Surgery

BMs from DTC tend to be highly destructive, resulting in pathological fractures and spinal cord compression from lesions in the spine. These SREs, including intractable pain and neurological symptoms, severely compromise the PS and QOL of patients. Local tumor control without SREs is important for patients with DTC-BMs because the prognosis of these patients is more favorable compared with that of patients with BMs from other cancers. Therefore, surgery for BMs is indicated more often than that for other organ metastases. For BMs, there are palliative and excisional surgery (metastasectomy) categories. Palliative surgery is performed to prevent symptomatic SREs, including pathological fractures and spinal cord compression, or to alleviate symptoms due to SREs. Metastasectomy is the complete excision of the tumor, aimed at achieving long-term local tumor control.

#### 3.1.1. Palliative Surgery (Stabilization with or without Partial Tumor Resection)

Osteolytic BMs from DTC easily cause SREs, especially in the spine and lower limb bones, which require weight bearing in daily activities [12]. Palliative surgery is mainly indicated in the presence of pathological or impending fracture risk and spinal cord compression with or without vertebral fracture [2]. In palliative surgery, reconstruction or fixation of the diseased lesion is the main procedure, and spinal cord decompression with partial resection of the tumor is also applied to the spinal lesion.

To aid clinicians in the diagnosis of neoplastic instability, an 18-point Spinal Instability Neoplastic Score (SINS) [39] for spinal lesions and a 12-point Mirels score [40] for upper and lower extremity lesions have recently been the most widely-used systems. The SINS system for the spine includes six parameters: location, pain, alignment, osteolysis, vertebral body collapse, and posterior element involvement. A high score, from 13 to 18, indicates the need for surgical stabilization to restore spinal stability from the affected lesion. The Mirels system for the extremities includes four parameters: location, pain, osteolysis, and tumor size. A high score, from 9 to 12, indicates the need for surgical intervention. These criteria have been shown to be valid, reliable, and reproducible [41,42].

#### 3.1.2. Metastasectomy (Complete Resection of the Tumor)

Generally, BMs from DTC are more resistant to radiotherapy and systemic therapy than other metastases [2,43]. A significant proportion of patients with DTC-BM in the spine, which is the site most affected by DTC-BM, have a solitary spinal lesion without non-spinal BMs or other organ metastases [5]. Based on these factors, skeletal lesions from DTC have the best indication for metastasectomy, if feasible. Surgery is intended to improve or maintain the QOL and PS over a long-term period and to prolong survival [5]. Since the 2000s, metastasectomy for DTC-BM has been reported to be a significant factor associated with improved survival rates [20,44,45]. The guidelines state that complete resection of BMs can prolong survival and is particularly appropriate for younger patients [1,46]. Moreover, the declining performance of daily activities and neurological deficits caused by BMs make it difficult for patients to undergo RAI therapy, which is the mainstay of treatment for metastases, especially in vital organ lesions, from DTC. Thus, metastasectomy of skeletal lesions, if achievable, should be considered. This aggressive surgery can be applied to patients with metastases from DTC because of its unique characteristics, mentioned above, and its favorable prognosis. The treatment strategy for thyroid BMs is therefore different from that for BMs from other malignancies.

Table 1 presents studies of surgery for BM from thyroid carcinoma, mainly DTC, with detailed clinical results, including information about postoperative survival and/or local tumor control in the operated lesions [9,47,48,49,50,51,52]. To reflect the most contemporary practice, only studies published in the last 10 years are included. However, there are few comparative studies on complete and incomplete excision of DTC-BM [9,47,48]. The postoperative survival rate of patients undergoing metastasectomy was more favorable, with lower local recurrence rates, than that of patients who underwent incomplete excision [47,48]. Kato et al. examined the minimum 4-year postoperative outcomes for patients who underwent surgery for spinal lesions and reported that only one patient who underwent complete excision experienced local tumor recurrence in the operated spine, whereas all long-term survivors (>18 months after surgery) in the incomplete excision group experienced local tumor recurrence and a consequent deterioration of PS [48]. Satcher et al. examined the clinical outcomes for patients who underwent surgery for appendicular skeletal lesions; after adjusting for age and sex, they reported that patients who had their tumor excised or presented with solitary bone involvement had a lower risk of death [49]. Yin et al. examined the clinical outcomes for patients with BMs in the cervical spine, which severely compromised the PS of the patients; they reported that the strongest factor in improved survival rates after the diagnosis of cervical spine metastasis was local disease control of the lesion, and that surgical intervention was significantly associated with improved survival [52].

**Figure 1:**
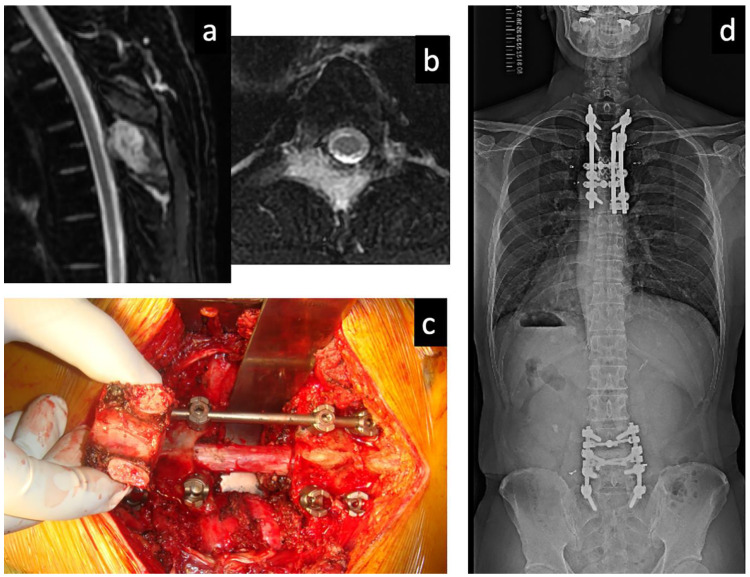
A 39-year-old man diagnosed with multiple lung and spinal metastases of T4 and L4. He underwent metastasectomies for spinal lesions. He also underwent radioactive iodine (RAI) therapy after the spinal metastasectomies and other metastasectomies for BMs, which subsequently appeared in the sacrum, left ilium, and humerus after RAI therapy. Eleven years after the first metastasectomy, he had no local tumor recurrences in the operated lesions; he still performed his normal daily activities and worked without any difficulties. (**a**) Sagittal and (**b**) axial T2-weighted magnetic resonance imaging of the thoracic spine, showing metastasis of T4. (**c**) Spondylectomy of T4 (complete resection of the tumor-affected vertebra) without any significant perioperative complications. (**d**) A recent full-spine radiography showing good maintenance of the reconstructed spine.

Excisional surgery for BMs, especially in the spine, is a remarkable and technically demanding surgery for general orthopedic and spine surgeons because the metastases are hypervascular and destructive, and reconstruction to support the operated lesion against load is required after tumor resection in most cases. Although it is not always feasible, complete resection of macroscopically identified bone tumor is recommended, and a favorable outcome has been reported even in patients with coexisting controlled lung metastases (Figure 1) [48]. Isolated and resectable BMs from kidney cancer are also indicated for metastasectomy. A simple and tailored treatment algorithm for spinal metastases from these two cancers has been reported [53], and it can be adapted for nonspinal BMs.

### 3.2. Radiotherapy

The main treatment goals for patients with BMs are symptom palliation and maintenance or improvement of PS and QOL. Conventional EBRT has been used as the primary and adjuvant treatment for BMs for decades. Recently, the demand for long-term local control of solitary or oligometastatic bone lesions, stereotactic radiosurgery (SRS), has become popular as the mainstay of treatment for long-term BM control.

#### 3.2.1. Conventional Radiation Therapy

EBRT is widely used as a local treatment for BMs. It can be used to complement surgery or alone in cases with intractable bone pain to reduce the pain and/or prevent pathological fractures, or in cases with spinal cord compression [54]. However, it is likely that conventional EBRT is related to a higher rate of relapse in patients who live longer. Although patients with mechanical instability in skeletal lesions require surgical stabilization, patients with low SINS or Mirels scores typically experience resolution of pain after radiotherapy [55,56]. EBRT generally delivers wide-field radiation in small additive doses, such as 30 Gy in 10 fractions. The dose is delivered to the tumor, although it is limited by the amount that can be tolerated by the surrounding organs at risk, such as the spinal cord.

Despite the relative radioresistance of DTC [57], EBRT is the main and standard treatment option for patients with symptomatic or asymptomatic BMs at a higher risk of fracture and/or neurological symptoms.

#### 3.2.2. Stereotactic Radiosurgery

The development of SRS, which can be used to deliver significantly high radiation doses with submillimeter accuracy, has changed the treatment paradigm, especially for patients with oligometastases, including BMs. It can deliver high-dose radiation (14–16 Gy in a single fraction) to the target volume, while sparing adjacent at-risk critical organs [58]. Owing to these characteristics, SRS can offer favorable outcomes and allow the re-irradiation of previously treated sites if necessary.

Recently, several studies have reported the efficacy of SRS for DTC-BM, although treatment protocols of SRS are different [59,60,61,62,63]. Table 2 presents studies of SRS for BM from thyroid carcinoma, mainly DTC, with detailed clinical results, including information about post-treatment survival rates and/or local tumor control in the treated lesions [59,60,61,63]. Bernstein et al. prospectively evaluated the efficacy of frame-based SRS in 23 patients with thyroid cancer, with 27 spinal lesions, as primary or adjuvant/salvage therapy. They reported that the local tumor control rates were 88% and 79% at 2 and 3 years, respectively. Pain flare was observed in 30% of patients in the median follow-up of 29 months [59]. Ishigaki et al. retrospectively evaluated the efficacy of SRS using the Cyberknife system and reported the local control rate of 97% at 1 year in 13 patients with DTC with 60 skeletal lesions, including only 7 symptomatic lesions [60]. Meanwhile, a recent retrospective study of 12 patients with 32 spinal lesions treated with Cyberknife reported a lower local tumor control rate of 67% at 1 year [61]. This difference between clinical outcomes could be due to the baseline characteristics of the BM lesions (a proportion of large and/or spinal lesions associated with significant symptoms and local tumor control). Another retrospective study reported that the use of Cyberknife SRS for DTC-BM was considered successful [62]. The largest series, including 67 patients and 133 skeletal lesions, reported excellent outcomes of 96% and 82% in 1- and 5-year local control rates, respectively [63].

In all the previously cited studies, SRS was effective and safe without the occurrence of spinal cord injury. However, a potential risk of vertebral compression fractures after treatment has been reported. Risk factors for fractures include older age, baseline fracture or pain, osteolytic lesion, higher tumor burden, higher radiation dose, and spinal deformity [64,65]. In patients with these risk factors and high SINS or Mirels scores, prophylactic stabilization should be considered before applying SRS to avoid the complication [64,65]. For patients with epidural disease, separation surgery focused on circumferential spinal cord decompression is performed to create an adequate distance (typically 1–2 mm) between the tumor and the spinal cord to safely provide optimal dosing in the following SRS [66,67].

SRS treatment is reported as showing a trend toward a significant improvement in PFS and OS rates in patients with oligometastatic disease from other cancers [68]. However, the effect of this treatment on survival rates among patients with DTC-BM remains unclear, in contrast to the effect of metastasectomy. A recent nationwide multicenter study has reported no significant effect of EBRT in decreasing the overall mortality of patients with DTC-BM [17]. Future studies are required to identify patients amenable to SRS and its effect on survival.

### 3.3. Percutaneous Procedures

Percutaneous procedures play an important role in the management of oligometastatic BMs from DTC. They are less invasive alternatives to surgery, especially in patients with decreased PS that is not suitable for surgery or with local tumor recurrence at the previously operated site. They can be applied in combination with systemic therapy in cases of symptomatic BM at a higher risk of local complications. The available percutaneous techniques for BMs from DTC are categorized into ablative, vascular, and consolidative treatment, which can be applied alone or combined and tailored according to the specific needs of the patient [69]. Cazzato et al. published their experience with percutaneous procedures including cementoplasty (77.5%) and ablation techniques (22.5%) for BMs from DTC. They reported a complete local remission rate of 56% at a median follow-up after treatment of 4.6 years, and an OS rate after treatment of 72%, 67%, and 60% at 1, 2, and 3 years, respectively [70]. However, well-designed studies of these techniques are scarce; most are retrospective, reliant on small sample sizes, and often conducted without a long-term follow-up. Future studies that compare the efficacy and tolerability of different procedures are required.

#### 3.3.1. Ablation Techniques

Thermal ablation techniques, including radiofrequency ablation and cryoablation, are minimally invasive treatments that create local tissue necrosis around the tip of a needle by heating or freezing the tissue, respectively. These therapies have also been applied in patients with DTC-BM [70,71]. Another ablation technique is microwave ablation, which uses electromagnetic waves to increase the intra-tumoral temperature. After the application of these ablation therapies for BM, consolidation with surgical or percutaneous techniques is required for the sites exposed to mechanical stress to avoid secondary pathological fractures [69]. Ablation techniques, which are available either alone or in combination with cementoplasty, are found to be effective and safe treatments for painful metastases [70]. Although thermal ablation techniques are usually used for palliation or for the prevention of symptoms from BM, in the selected patients they have a potentially curative role, which should be further explored and which can be advanced in the future [72].

#### 3.3.2. Cementoplasty

Percutaneous cementoplasty (vertebroplasty in the spine) is a minimally invasive procedure that involves the injection of bone cement (polymethylmethacrylate) into BMs with structural weakness, to provide pain relief and mechanical stability [73,74]. This procedure is usually applied to patients experiencing significant pain due to osteolytic and destructive BMs, especially in weight-bearing bones, including the spine and pelvis, which are common sites for DTC-BM [70,74,75]. Cementoplasty can be used in combination with other procedures, such as radiofrequency ablation and RAI therapy [75]. A careful indication of cementoplasty is required in patients with solitary or oligometastatic lesions because the procedure can theoretically increase the number of circulating tumor cells from the treated BMs [76]. A case report has demonstrated that pulmonary intravascular metastases developed as a result of vertebroplasty for prostate cancer spinal metastases [77].

#### 3.3.3. Embolization

Percutaneous transarterial embolization has been widely applied for the treatment of BMs from DTC alone or in combination with other treatments [69]. This technique aims to provide devascularization and size reduction of the tumor tissue through vascular occlusion by several embolic materials, causing ischemia and subsequent necrosis. The efficacy of the procedure for BMs from DTC is related to the hypervascularity’s characteristics. The procedure alone can provide palliation or the prevention of symptoms and reduce tumor burden for more than half of patients [78]. However, its efficacy is usually rapid, but transient. The procedure is often performed just before surgery to reduce operative bleeding, shrink tumor size, and allow a clearer separation between the tumor and the surrounding tissues [79,80]. The combination of EBRT and RAI therapy has a potential effect on the prolonged duration of symptom control without tumor progression [81].

## 4. Conclusions

Patients with BMs, especially those who have them in the spine, have a worse prognosis than those with lung metastasis in multiple cancer subtypes. However, the prognosis of patients with BM from DTC is still favorable compared to that of patients with other cancers. Patients with osteolytic BMs from thyroid carcinoma often have a significantly reduced PS. The PS affects mortality directly and indirectly by hampering the application of systemic therapies using RAI and/or KIs, which are the mainstay of treatment for patients with metastatic DTC. Therefore, long-term local control of BMs from DTC is desirable, especially in patients with single or oligometastases. Along with systemic therapies, local therapies, including metastasectomy and SRS, can be valuable as treatment options, and even as curative measures of BM in selected patients. Recent advancements in local therapies have the potential to provide not only long-term local tumor control but also a better prognosis.

## Figures and Tables

**Table 1:** Clinical outcomes of surgery for thyroid cancer BMs published in the last 10 years.

Study [Ref. No.] (Year of Pub.)	No. of Patients (M/F)	PTC/ FTC/ Others	Mean Age (Years; Range)	Location	Surgery (No.)	Mean Follow-Up after Surgery (Mos; Range)	5-Year Survival Rate after Surgery (Median Survival)	Local Tumor Control in the Operated Spine
**Three studies including the detailed outcomes of complete excision (metastasectomy for BM from thyroid carcinoma)**								
Demura S [47] (2011)	24	8/15/1	60.7 (39–77)	Spine: 24	Complete Ex: 10 Incomplete Ex: 14	55 mos (12–180)	All: 74%, Complete Ex: 90%, Incomplete Ex: 63%	[LR rate] Complete Ex: 10% Incomplete Ex: 57%
Nakayama R [9] (2014)	40 (16/24)	12/28/0	40.6 (23–64)	Spine: 18 Nonspinal bone: 34	Complete Ex: 35 Incomplete Ex: 17	46 mos [median] (4–233)	All: 64%	[5-year LC rate] Complete Ex: 84% Incomplete Ex: 55%
Kato S [48] (2016)	32	10/21/1	60.5 (N/A)	Spine: 32	Complete Ex: 20 Incomplete Ex: 12	N/A (>4-year post-op FU)	All: 71%, Complete Ex: 84%, Incomplete Ex: 50%	[LR rate] Complete Ex: 5% Incomplete Ex: 75%
**Four studies detailing the outcomes of surgery for BM from thyroid carcinoma**								
Satcher RL [49] (2012)	41 (19/22)	21/6/14	59 (12–82)	Nonspinal bone: 41	Complete Ex: 15 Incomplete Ex: 19 No Ex: 7	60 mos [median] (10–102)	29% (22.8 months)	LR rate: 20%
Sellin JN [50] (2015)	43	9/20/14	59 (36–79)	Spine: 43	Incomplete Ex: 43	39 mos (2–63) for 4 patients who were alive at last FU	N/A (15.4 months)	N/A
Zhang D [51] (2019)	52 (17/35)	7/43/2	57.6 (26–82)	Spine: 52	Complete Ex: 8 Incomplete Ex: 44	47 mos (12–126)	79%	N/A
Yin LX [52] (2020)	16	8/4/4	66 (at last FU)	Cervical spine: 16	Incomplete Ex: 16	30 mos (after diagnosis of BM)	45% (after diagnosis of BM)	N/A

BM, bone metastasis; Ex, excision; F, female; FTC, follicular thyroid carcinoma; FU, follow-up; LC, local control; LR, local recurrence; M, male; N/A, not available; No., number; PTC, papillary thyroid carcinoma; pub., publication.

**Table 2:** Four studies that included detailed outcomes of SRS for BM from thyroid carcinoma.

Study [Ref. No.] (Year of Pub.)	No. of Patients (M/F)	PTC/ FTC/ Others	Median Age (Years; Range)	Location	SRS Characteristics	Median Follow-Up after SRS (Mos; Range)	Survival Rate after SRS (Median Survival)	Local Tumor Control Rate in the Treated Lesions
Bernstain MB [59] (2016)	23 (13/10)	9/6/8	58 (33–79)	Spine: 27	16–18 Gy in 1 fr 27–30 Gy in 3 to 5 fr	29 mos (5–93)	85% and 67% at 1 and 2 years, respectively	88% and 79% at 2 and 3 years, respectively
Ishigaki T [60] (2019)	13 (3/10)	3/9/1	69 (42–87)	Spine: 28 Nonspinal bone: 32	8–48 Gy in 1–10 fr (median; 27 Gy, 3 fr)	11 mos (2–56) in 40 lesions that were assessable for effectiveness	75% and 38% at 3 and 4 years, respectively	97% at 1 year
Hariri O [61] (2019)	12 (8/4)	5/6/1	71 (48–87)	Spine: 32	Mean dose: 20 Gy given in 1 to 4 fr	29 mos (0.5–140) 17 mos for imaging evaluation	55%, 44%, and 33% at 1, 2, and 3 years, respectively	67%, 56%, and 34% at 1, 2, and 3 years, respectively
Boyce-Fappiano D [63] (2020)	67 (34/33)	22/24/21	60 (28–80)	Spine: 133	18–24 Gy in 1 fr 27–30 Gy in 3–5 fr	31 mos for patients who were alive at last FU	86%, 74%, and 44% at 1, 2, and 5 years, respectively (43 mos)	96%, 89%, and 82% at 1, 2, and 5 years, respectively

BM, bone metastasis; DTC, differentiated thyroid carcinoma; F, female; Fr, fraction; FTC, follicular thyroid carcinoma; FU, follow-up; Gy, gray; LC, local control; LR, local recurrence; M, male; N/A, not available; No., number; PTC, papillary thyroid carcinoma; pub., publication; SRS, stereotactic radiosurgery.
